# A discussion on ‘Domain formation and phase transitions in the wurtzite-based heterovalent ternaries: a Landau theory analysis’

**DOI:** 10.1107/S2053273321001376

**Published:** 2021-03-23

**Authors:** Paul C. Quayle, Joachim Breternitz

**Affiliations:** a Department of Electrical and Computer Engineering, Michigan State University, 428 South Shaw Lane, East Lansing, Michigan 48824-1226, USA; b Structure and Dynamics of Energy Materials, Helmholtz-Zentrum Berlin für Materialien und Energie, Hahn-Meitner-Platz 1, 14109 Berlin, Germany; c Institute for Chemistry, Universität Potsdam, Karl-Liebknecht-Strasse 24/25, Potsdam, 14476, Germany

**Keywords:** group–subgroup relationships, nitride materials, wurtzite type

## Abstract

Heterovalent ternary nitrides are considered one of the promising classes of materials for photovoltaics, combining attractive physical properties with low toxicity and element abundance. One of the front-runner systems under consideration is ZnSnN_2_. Although it is nominally a ternary compound, no clear crystallographic evidence for cation ordering has been observed so far. An attempt to elucidate this discrepancy [Quayle (2020). *Acta Cryst.* A**76**, 410–420] was the trigger for an intensive discussion between the authors, and an agreement was reached to elaborate on some points in order to set things in perspective. Rather than using a conventional comment–answer scheme, this is published in the form of a joint discussion to celebrate constructive criticism and collegiality.

## Abstract 2

A scientific exchange on an earlier paper [Quayle (2020). *Acta Cryst.* A**76**, 410–420] has led to the clarification of some of the points.

## 1. How it all started   

Shortly after the publication of Quayle (2020 ▸), I received a message titled ‘On: ‘Domain formation and phase transitions in the wurtzite-based heterovalent ternaries: a Landau theory analysis’ in which Joachim Breternitz pointed out, in the most courteous and professional manner, an error in the proposed Landau theory. What followed was an exchange concerning the nature of atomic disorder in the wurtzite-based ternaries; an exchange that has led us to a satisfying impasse. We have arrived at two distinct, seemingly plausible positions regarding ternary disorder, and we report here on our discussion in the hope that insights from the research community will lead to a resolution.

That there was an error in the Landau theory in Quayle (2020 ▸) is not in dispute. As discussed later, Quayle (2020 ▸) treats two different crystal structures, both members of the same space-group type but with different unit-cell sizes and atom locations, as one and the same, and the Landau theory built from this oversight is flawed. A straightforward fix to the analysis is achieved through the correct assignment of the two structures; however, resolving that issue introduces a disagreement between the theory and relevant experimental observations, throwing doubt on the methodology as a whole. This is the heart of the matter. Can the mismatch between the formally correct analysis and experimental observations be rectified? Or is the analysis logically sound, but not consistent with nature?

## 2. Introduction   

Binary III–V nitrides in the wurtzite-type structure are candidate materials for photovoltaic applications (Jani *et al.*, 2007 ▸); however, indium and gallium are rather scarce and expensive elements, and more earth-abundant alternatives are sought-after for low-cost applications. The II–IV–N_2_ compounds are a promising class of materials which are formally derived from III–V materials by replacing the tri­valent cation in the binary compound by a stoichiometric mixture of divalent and tetravalent cations. With a number of cations potentially filling the divalent (*e.g.* Zn^2+^, Mg^2+^, Cd^2+^) and tetravalent (Si^4+^, Ge^4+^, Sn^4+^) positions, the chemical parameter space allows wide variations that in turn affect the bandgap and enable bandgap tuning (Martinez *et al.*, 2017 ▸). However, in addition to mixing different tetravalent cations occupying the same sites, these materials are expected to show a second mechanism of bandgap tuning through order/disorder phenomena of the cations (Quayle *et al.*, 2015 ▸).

ZnSnN_2_ is distinguished from its sister ternary compounds. ZnSnN_2_ is an earth-abundant semiconductor that has a bandgap very much in the ideal range for photovoltaics (Veal *et al.*, 2015 ▸). Further, it has demonstrated distinct disorder properties; to date, there have been no reported observations of ordering in ZnSnN_2_, even in material that emits light at room temperature (Quayle *et al.*, 2015 ▸). Density functional theory (DFT) calculations suggest that the bandgap of ZnSnN_2_ can be lowered by 0.85 eV in fully disordered mater­ial, with a randomized cation sublattice, compared with perfectly ordered material with the β-NaFeO_2_-type structure (Makin *et al.*, 2019 ▸).

The wurtzite-type structure crystallizing in space group *P*6_3_ *mc* possesses only one independent cation position and hence does not allow for ordering of the heterovalent ternary cations (Breternitz *et al.*, 2019 ▸). To find a crystallographic description compatible with ordered cations, one must follow a descent into symmetry (*e.g.* Quayle, 2020 ▸; Breternitz & Schorr, 2021 ▸). Indeed, most of the experimentally observed ternary nitrides crystallize in the orthorhombic space group *Pna*2_1_ in a β-NaFeO_2_-type structure (β-*Pna*2_1_), which contains two crystallographically independent cation positions (Breternitz & Schorr, 2021 ▸). The experimental observations agree with computational physics results which calculate that the β-*Pna*2_1_ is the lowest-formation-energy phase at 0 K. The other possible ordered phases can have close formation energies however, and this is especially true in the case of ZnSnN_2_. The difference in formation energy of ZnSnN_2_ between the β-*Pna*2_1_ and *Pmc*2_1_ structures was calculated to be only 0.01 eV per formula unit (Quayle *et al.*, 2015 ▸). The similar formation energies of the phases coupled with the lack of ordering observed experimentally raise the question of whether a mixture of phases can be present on the ZnSnN_2_ lattice, and whether the phase composition can change as temperature is raised towards the melting point. Indeed, phase transitions are widely reported in the zincblende-based chalco­pyrite heterovalent ternaries with stoichiometries II–IV–V_2_ and I–III–V_2_ (Zunger, 1987 ▸). Another candidate atomic arrangement is an essentially randomized cation substructure, which keeps perhaps a certain degree of local bond order, but which is isotropic on the more macroscopic scale.

Quayle (2020 ▸) set out to explore a phase transition and the ordering question in ZnSnN_2_ from the angle of the Landau theory. This theory is grounded on the analysis of structural similarities of different crystal structures and the identification of ordering parameters based on these similarities. The purpose of this paper is to clarify some details of the Landau theory and introduce additional considerations elucidated in the ‘prequel’ paper by Breternitz & Schorr (2021 ▸).

## 3. The group–subgroup relation between β-NaFeO_2_- type in space group *Pna*2_1_ and the structure in space group *Pmc*2_1_   

**Figure 1:**
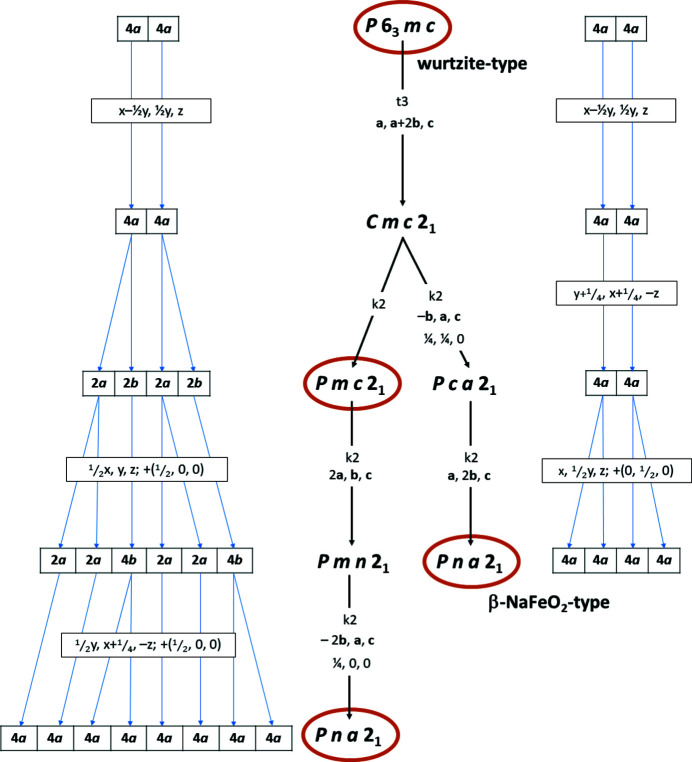
Group–subgroup relationship between the relevant subgroups of the wurtzite type (*P*6_3_ *mc*). The *Pmc*2_1_ structure, the derived hypothetical *Pna*2_1_ structure (α-*Pna*2_1_) and the β-NaFeO_2_-type structure (β-*Pna*2_1_) are highlighted for visibility. Further, the Wyckoff positions and transitions of the atomic positions are given.

A group–subgroup relationship between the hypothetical *Pmc*2_1_ structure (in the following simply abbreviated as *Pmc*2_1_) and a crystal structure with space group *Pna*2_1_ is at the core of the argument by Quayle. This latter is implicitly taken as the observed β-NaFeO_2_-type structure. The two phases discussed by Quayle, the hypothetical *Pmc*2_1_ structure and the β-NaFeO_2_-type structure (which we further denote as β-*Pna*2_1_ for simplicity), are clearly both subgroups of the wurtzite type (Breternitz & Schorr, 2021 ▸). However, they do not lie on the same branch of the Bärnighausen tree (Bärnighausen, 1980 ▸), in which the group–subgroup relations are outlined graphically (Fig. 1 ▸). In fact, β-*Pna*2_1_ is not a maximal subgroup of *Pmc*2_1_. The group–subgroup relationship between *Pmc*2_1_ and a structure in space group *Pna*2_1_ that is established by Quayle (2020 ▸) through the intermediate space groups *Pmn*2_1_, *Pca*2_1_ or *Cmc*2_1_ (the latter two alternatives not depicted in Fig. 1 ▸) is, nonetheless, formally valid. The transition path described does, however, end up in a unit cell that is twice as large as the one of β-*Pna*2_1_ and is therefore *not equivalent*. For the sake of readability, we will further denote this hypothetical crystal structure as α-*Pna*2_1_.

We also note that all three symmetry descents lead to equivalent solutions. The statement ‘The *Cmc*2_1_ phase is not compatible with the orthorhombic crystal structure’ by Quayle (2020 ▸) is a common misinterpretation of *klassengleiche* phase transitions: the volume of the intermediate *Cmc*2_1_ is four times that of *Pmc*2_1_ but the latter is still a k2 subgroup, because of the introduction of centring in this symmetry transition. In return, the subsequent k2 descent from *Cmc*2_1_ to α-*Pna*2_1_ does not cause a volume increase, but instead is achieved through the loss of centring.

It really needs to be highlighted that the interpretation of group–subgroup relationships can be a complicated and tedious path and we would like to take this opportunity to give some guidance on the indicators that can act as a ‘safety net’ insofar as they may point to oversights. Firstly, it is crucially important to make the distinction between space groups and space-group types, two terms that are often used synonymously. From a purely qualitative point of view, one may view the difference as follows: the 230 space-group types define all possible arrangements of the symmetry elements in three-dimensional crystal structures and their *relative* position to each other (*e.g.* the origin versus the unit-cell centre). A space group, on the other hand, can be understood as defining the *concrete* arrangement of the symmetry elements, or in other words, a space group also requires the unit-cell dimensions. A more formal definition may be found in sections 8.1.6 and 8.2.2 of Volume *A* of the *International Tables for Crystallography* (Hahn, 2005 ▸). If the (approximate) unit cells of the crystal structure derived from a group–subgroup relationship do not coincide with those determined experimentally, this should indeed raise serious concerns about the validity. Secondly, the atomic positions of the group–subgroup relationship derived crystal structure must be in line with those that are experimentally observed. We note that this point is even more complex in this case, as the atomic positions in the crystal structures in either *Pna*2_1_ all lie on general positions (Wyckoff position 4*a*), which means that the choice of origin within the unit cell is arbitrary. In such a case, it is best either to use the relative relationships between the atomic positions, or to apply an origin shift to one of the two structures, so that they overlap.

## 4. How do the crystal structures compare?   

**Figure 2:**
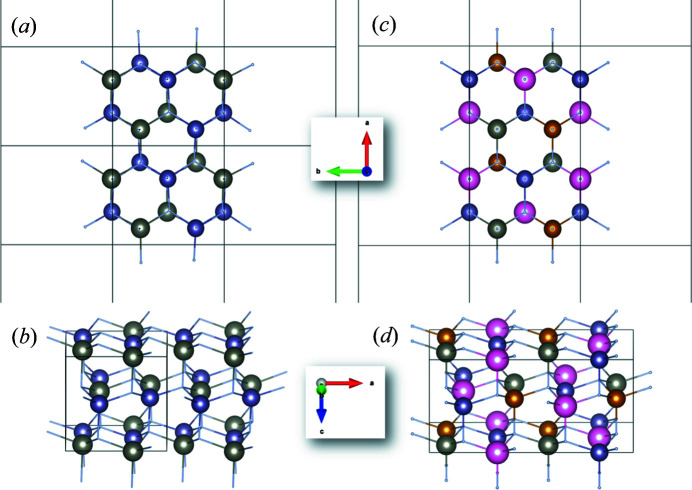
Structural representations of the β-*Pna*2_1_ (*a*), (*b*) and the hypothetical α-*Pna*2_1_ structure (*c*), (*d*). Views are along the crystallographic *c* axis (*a*), (*c*) and as a general view (*b*), (*d*). All crystallographically independent cation positions are highlighted as large spheres in different colours, while the nitro­gen atoms are very small to increase visibility of the cations. The crystal structure of the α-*Pna*2_1_ structure was constructed from the wurtzite type using the *TRANSTRU* tool of the Bilbao Crystallographic server (Aroyo, Perez-Mato *et al.*, 2006 ▸; Aroyo, Kirov *et al.*, 2006 ▸; Aroyo *et al.*, 2011 ▸).

The α-*Pna*2_1_ structure derived as a hettotype of the *Pmc*2_1_ structure has four independent crystallographic positions for the cations and four for the anions, while β-*Pna*2_1_ has only two sites for cations and anions, respectively (Fig. 1 ▸). Given the higher number of independent crystallographic positions in α-*Pna*2_1_, it seems possible that one could represent the β-*Pna*2_1_ structure as α-*Pna*2_1_. To do this, one must establish a group–subgroup relationship between the two types, so that the α-*Pna*2_1_ structure would be a common subgroup of *Pmc*2_1_ and β-*Pna*2_1_. In fact, Baur & McLarnan (1982 ▸) did explicitly draw the corresponding *isomorphous* symmetry descent of index 2 through a doubling of the *a* axis in their seminal work on symmetry relationships in wurtzite materials. However, this is, unfortunately, incorrect, as no *isomorphous* descent of index 2 is possible in this space-group type, but only of index 3 or larger: the *n*-glide planes perpendicular to the *a* axis are situated at *a* = ¼ and *a* = ¾ in space-group type *Pna*2_1_. If a doubled *a*-axis *a*′ is considered (as for an i2 descent), the *n*-glide plane lying at *a*′ = ¼ in this space group would have to be situated at *a* = ½ in the smaller unit cell, but this is not compatible with the symmetry in space-group type *Pna*2_1_. If one triples the *a* axis, on the other hand, the *n*-glide plane at *a* = ¼ is compatible with the one at *a* = ¾. From a more visual point of view, it becomes evident that α-*Pna*2_1_ and β-*Pna*2_1_ cannot be linked through a direct group–subgroup relationship, since their ordering motifs are different (Fig. 2 ▸). As a matter of fact, there is no possible ordering of cations in the α-*Pna*2_1_ structure that would fulfil the β-*Pna*2_1_ structure, as would be a prerequisite for a group–subgroup relationship.

There is another aspect of this story that deserves consideration, which comes from molecular dynamics (MD) simulations. Lany *et al.* (2017 ▸) reported on simulation results of possible structures of ZnSnN_2_ that obey Pauling’s rules, and they calculated the total energy of the α-*Pna*2_1_ structure to be between the thermodynamically stable β-*Pna*2_1_ structure and the *Pmc*2_1_ structure. This result demonstrates two points: (i) the α-*Pna*2_1_ and β-*Pna*2_1_ structures are distinctly different, not only from a structural point of view, but also from their properties; (ii) their differences are probably not so large that the ordering of the α-*Pna*2_1_-type structure would be completely implausible.

**Figure 3:**
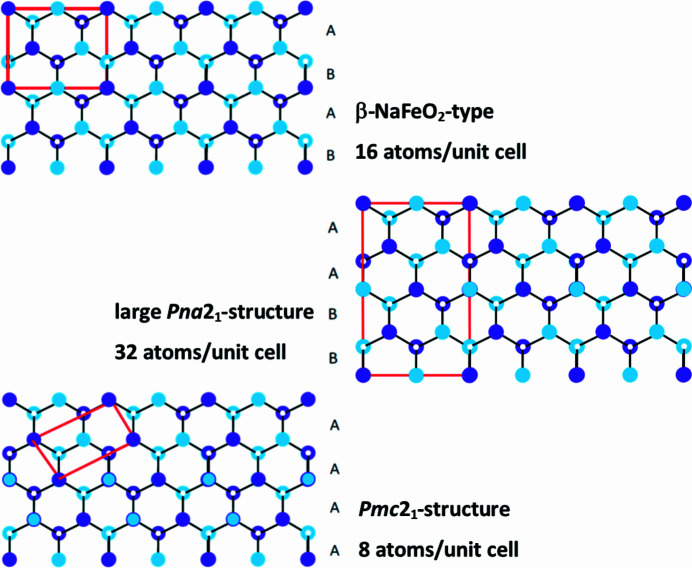
Comparison of the stacking motifs in the different observed and hypothetical crystal structures for ZnSnN_2_.

Fig. 3 ▸ shows the β-*Pna*2_1_ and α-*Pna*2_1_ structures again along with the *Pmc*2_1_ structure from a different perspective. When viewed along the polar *c* axis, we see that the structures consist of different stacking motifs of the same *c*-axis basal plane (Quayle *et al.*, 2015 ▸). The β-*Pna*2_1_ structure consists of two distinct motifs (*A* and *B*), while *Pmc*2_1_ consists only of one. In other words, the β-*Pna*2_1_ structure is built from an *ABAB* stacking of the basal plane while the *Pmc*2_1_ structure consists of an *AAAA* stacking. The α-*Pna*2_1_ structure consists of both stacking motifs, *AABB*. When viewed in this way, it is not surprising that the calculated formation energy of the α-*Pna*2_1_ phase is intermediate between the *Pmc*2_1_ and β-*Pna*2_1_ phases.

## 5. What are the implications for the physical model?   

The symmetry relation upon which the Landau theory of Quayle (2020 ▸) is based is between the *Pmc*2_1_ structure and the α-*Pna*2_1_ structure; therefore, the derived order parameter describes this phase transition. Quayle (2020 ▸) however describes the transition, and order parameter, as between the *Pmc*2_1_ structure and the β-*Pna*2_1_ structure. What are the implications of this error?

The analysis of Quayle (2020 ▸) is, in essence, a thought experiment to explain the discrepancy between the β-*Pna*2_1_ being reported to be the thermodynamically stable crystal structure – which also is observed for the lighter analogue ZnGeN_2_ – and the X-ray diffraction (XRD) results of ZnSnN_2_ adopting a wurtzite-type structure (*e.g.* Feldberg *et al.*, 2012 ▸; Quayle *et al.*, 2013 ▸; Kawamura *et al.*, 2016 ▸).^1^ The question is whether this observation is due to the fact that the different cations are truly randomly distributed, or whether they still follow certain patterns on a local level. Given the limitation of the diffraction method as averaging over space and time, both cases could result in virtually identical diffraction patterns.

In Quayle (2020 ▸), we picture the situation in which a solid-state phase transition occurs between a *Pmc*2_1_ group phase, which is more stable at high temperatures, and a β-*Pna*2_1_ ‘subgroup’ phase, which is more stable at low temperatures. In a hypothetical *Pmc*2_1_ phase, with perfect crystallinity, as the temperature is lowered towards and below the transition point, the β-*Pna*2_1_ phase precipitates and grows to become the dominant phase. In Quayle (2020 ▸), it was proposed that the transition is from a paraelectric *Pmc*2_1_ phase to an anti­ferroelectric β-*Pna*2_1_ phase. Now, we see from the analysis given above that the order parameter utilized in Quayle (2020 ▸) actually describes the transition from *Pmc*2_1_ to α-*Pna*2_1_. For the sake of completeness, it needs to be noted that this is a thought experiment insofar as no such phase transition has been observed experimentally to date.

The misidentification of the order parameter is problematic because the α-*Pna*2_1_ phase is not the most energetically favourable phase, according to calculations, and the α-*Pna*2_1_ phase has never been observed experimentally in analogue ternary compounds, which have only exhibited signatures of the β-*Pna*2_1_ phase. The analysis is formally correct however if we realize that the subgroup phase is α-*Pna*2_1_ not β-*Pna*2_1_. Also, it can be argued that both *Pna*2_1_ phases are antiferroelectric, using the methodology in Quayle (2020 ▸), so that aspect of the analysis remains unchanged.

Still, for the Landau theory to truly describe nature, it must lead to a β-*Pna*2_1_ stable phase on the low-temperature side of the transition, and the analysis in Quayle (2020 ▸) does not achieve this. The formal analysis ends at the transition from *Pmc*2_1_ to α-*Pna*2_1_. To find a logical path from the α-*Pna*2_1_ phase to β-*Pna*2_1_ we must depart from group theory arguments and note that if a ternary nitride can transition from *AAAA* basal plane ordering (*Pmc*2_1_) to *AABB* ordering (α-*Pna*2_1_) by way of a mechanism similar to the one described in Quayle (2020 ▸), then it is plausible that it can also transition further to the more energetically favourable *ABAB* ordering of β-*Pna*2_1_.

A broader issue is raised by questioning the validity of the Landau theory. The *Pmc*2_1_ structure is probably a very good approximation to a Pauling rule conserving wurtzite-derived structure. This may sound contradictory at first, but as outlined in Breternitz & Schorr (2021 ▸), a main feature of the hypothetical structure in *Pmc*2_1_ is the fact that the symmetry of the space group prevents the tetrahedra around the cations from being different in size, which casts doubt on whether this system would profit from cation ordering in the first place. Given that all three structures under discussion were calculated to have very similar total energies (Lany *et al.*, 2017 ▸), this alone may be taken as a hint that cation ordering would not be greatly energetically beneficial in this system.

Both possible explanations for the observed wurtzite-type structure – a completely random cation distribution and a partial cation ordering on the local scale – would intrinsically have to produce some cation arrangements violating Pauling’s rules: in the first case these would be randomly distributed and in the second case these would reside at the interfaces between differently ordered domains.

Which of the two potential solutions, if one of them, is the one best describing reality is a question that merits further experimental studies using more local probes, such as electron diffraction and/or pair-distribution-function analyses and, potentially, spectroscopic studies, as the differences may be expressed in different optoelectronic and/or electronic properties of the observed macroscopic materials.

## Footnotes

1 We note that some publications (*e.g.* Feldberg *et al.*, 2012 ▸) also report monoclinic unit cells, but they are within the error of the hexagonal wurtzite type and there is hence little ground for this assumption.
